# Exploring parental and professional perceptions of weight management services among under-fives in England: A qualitative study

**DOI:** 10.1016/j.obpill.2024.100149

**Published:** 2024-11-13

**Authors:** Maines Msiska, Lawrence Achilles Nnyanzi, Misheck Julian Nkhata, Scott Lloyd, David B. Olawade

**Affiliations:** a School of Health & Life Sciences, Teesside University, United Kingdom; b NIHR Health Determinants Research Collaboration South Teesside, United Kingdom; c Department of Allied and Public Health, School of Health, Sport and Bioscience, University of East London, London, United Kingdom; d Department of Research and Innovation, Medway NHS Foundation Trust, Gillingham, ME7 5NY, United Kingdom; e School of Health and Care Management, Arden University, Arden House, Middlemarch Park, Coventry, CV3 4FJ, United Kingdom

**Keywords:** Childhood obesity, Weight management, Parental engagement, Cultural beliefs, Public health, Qualitative analysis

## Abstract

**Background:**

Childhood obesity is a significant public health challenge, particularly among children under five. In England, weight management programs aim to address this issue; however, engagement and uptake of these services remain low. Understanding the perceptions of parents and professionals involved in these programs is crucial to enhancing participation and improving health outcomes. This study explores these perceptions to identify barriers and facilitators to effective engagement with weight management services.

**Method:**

This qualitative study used purposive sampling to recruit parents and professionals involved in weight management programs across England. Semi-structured interviews were conducted with 15 participants (8 parents and 7 professionals). Data were transcribed, coded, and thematically analyzed using Braun and Clarke's six-phase framework to identify key themes related to engagement, cultural influences, and program effectiveness.

**Results:**

The study identified several key themes: parental engagement, the impact of virtual versus face-to-face sessions, motivations for participation, barriers to involvement, and the role of cultural beliefs. Parental involvement was critical for the success of weight management programs, but barriers such as time constraints, cultural beliefs, and socioeconomic factors hindered participation. Professionals highlighted the need for more training and resources to effectively address these challenges. Cultural sensitivities and systemic support were found to be crucial for improving engagement and outcomes.

**Conclusions:**

The findings emphasize the need for culturally tailored, accessible, and sustainable weight management interventions. Enhanced professional training, increased community outreach, and policy-level support are essential to improve engagement and ensure long-term success in addressing childhood obesity.

## Highlights

• Professionals report a need for more training and resources in weight management.
• Virtual vs. face-to-face sessions impact participation and program effectiveness.
• Socioeconomic and cultural beliefs influence engagement with obesity services.
• Study highlights gaps in current professional training for obesity management.

## Graphical abstract

**Figure:**
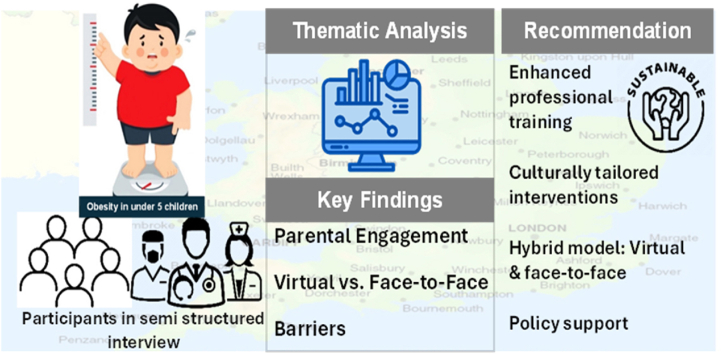

## 1 Introduction

Childhood obesity has emerged as a significant public health issue, posing a global crisis with increasingly alarming trends in many countries, including England. The World Health Organization (WHO) recognizes childhood obesity as one of the most serious public health challenges of the 21st century [1]. This condition extends beyond excess weight and involves multiple factors, including genetic predispositions, behavioral patterns, and socio-environmental influences [2]. In particular, obesity in early childhood (under five years of age) serves as a strong predictor of continued obesity into adolescence and adulthood. As a result, it becomes a critical determinant of chronic health conditions such as type 2 diabetes, cardiovascular diseases, and certain cancers later in life [3,4]. Moreover, the psychological consequences of obesity, including stigmatization, low self-esteem, and depression, compound the issue, making early intervention not just a medical necessity but a broader social priority [5]. The early years of a child's life represent a key developmental phase where dietary habits, physical activity patterns, and health behaviors are established. These formative years offer a window of opportunity for intervention, as the habits developed during this period significantly impact a child's future health trajectory [6]. Once obesity is established, it becomes more challenging to reverse; thus, early intervention in weight management is critical [7]. Public health initiatives, especially those targeting early childhood, are essential in preventing the escalation of obesity rates.

In England, various weight management services have been implemented to support children and their families in maintaining a healthy weight [8]. These services typically involve a multidisciplinary approach, combining nutritional guidance, promotion of physical activity, and behavioral support. However, despite these initiatives, engagement among parents of children under five remains low. The effectiveness of these interventions is highly dependent on parental involvement, as they play a pivotal role in shaping children's eating and activity habits. Without adequate participation from parents, the potential benefits of these weight management programs are significantly diminished [[9], [10], [11]]. Despite the availability of weight management services in England, a notable gap exists in the engagement and uptake of these services for children under five [12,13]. This underutilization poses a significant public health concern, as early intervention is essential in preventing the long-term consequences of childhood obesity. Several factors may contribute to this gap, including parental perceptions of healthy weight, cultural beliefs, socioeconomic status, and the effectiveness of communication from health and social care professionals. Parental understanding of weight issues can often differ from clinical definitions, with some parents viewing a slightly overweight child as healthy, particularly in younger children.

Health and social care professionals face their own challenges in addressing these issues. Approaching the sensitive topic of a child's weight without causing offense or distress can be difficult, and some professionals may feel ill-equipped to engage parents in meaningful discussions about weight management. These combined factors hinder the uptake of weight management services, limiting the potential impact of these programs. Addressing this issue requires a comprehensive understanding of the barriers and facilitators to service engagement, which this study aims to provide. Understanding the perceptions of both parents and professionals within the health and social care sectors is crucial to improving the uptake and effectiveness of weight management services [14]. Parents' perceptions of healthy weight, shaped by cultural, social, and environmental influences, greatly impact their willingness to engage with available services [15,16]. Health and social care professionals, on the other hand, play a crucial role in facilitating access to these services and encouraging healthy behaviors. However, their approaches and perceptions may differ, affecting how effectively they communicate the importance of weight management to parents.

Given the serious health risks associated with obesity, including its persistence into adulthood, there is an urgent need to enhance the effectiveness of early interventions. This study seeks to uncover the root causes of underutilization and identify strategies that can improve engagement, ultimately contributing to the development of more effective public health interventions. The rationale behind this study is based on the pressing need to address the low engagement rates with weight management services for children under five in England. This study aims to explore the perceptions of both parents and professionals regarding unhealthy weight in children under five years old, particularly in the context of their engagement with weight management services in England. By exploring the perceptions of both parents and professionals, this study provides insights that can help tailor weight management services to better meet the needs of those they aim to serve. In doing so, it aims to bridge the gap between the availability of these services and their utilization, leading to improved health outcomes for children at risk of unhealthy weight. By delving into the beliefs, concerns, and motivations of these two groups, the study identifies the factors influencing service uptake and engagement. The findings could inform future policies and interventions aimed at improving participation in weight management programs, ultimately contributing to better health outcomes for children at risk of unhealthy weight.

## 2 Methodology

### 2.1 Study design

This study employed a qualitative design, combining semi-structured one-to-one interviews with thematic analysis to explore the perceptions of parents and health/social care professionals regarding unhealthy weight in children under five years and their engagement with weight management services. Purposive sampling was used to identify participants with direct experience in either receiving or delivering weight management interventions for children. Telephone and Microsoft Teams were the primary mediums for conducting the interviews. All interviews were recorded, transcribed, coded, and analyzed thematically to uncover key themes and insights regarding barriers, facilitators, and perceptions around childhood obesity.

### 2.2 Study site

The initial study site was North East England, where ethical approval was granted for data collection. However, due to challenges in recruiting a sufficient number of participants, the study was expanded to include Medway, a borough in Kent, South East England, and the South Tees area, which includes Middlesbrough, Redcar, and Cleveland in North East England. This amendment facilitated a broader recruitment base, allowing the study to capture regional variations in perceptions and experiences related to childhood weight management. The North East of England has significant socioeconomic challenges, including low GDP per capita, high levels of unemployment, and some of the highest childhood obesity rates in England [17,18]. In contrast, Medway, while located in the more affluent South East of England, has childhood obesity rates closer to those in deprived areas like North East England [19]. These regions were selected to provide diverse socioeconomic and geographic contexts, essential for understanding the broader landscape of childhood obesity in England.

### 2.3 Study population

The study population included two distinct groups: parents of children under five years old and professionals (health and social care) involved in weight management services. The target population was carefully defined to ensure relevance and specificity to the research objectives [20]. This precise definition was crucial for collecting relevant data and enhancing the validity of the study findings [21].

### 2.4 Selection criteria and sampling technique

Participants included parents or caregivers of children under five years of age with an unhealthy weight, specifically those with a BMI greater than the 85th percentile for age, gender, and height. Professionals involved in advising, referring, commissioning, or delivering weight management services for children under five were also recruited. Children with a BMI within the healthy weight range (greater than the 5th percentile and less than the 85th percentile) were excluded, as were those with a BMI at or below the 5th percentile, indicating underweight status for age, gender, and height. This exclusion ensured that the study remained focused on the population at risk for unhealthy weight without confounding by different health etiologies related to underweight or healthy weight ranges.

Purposive sampling was employed to ensure that participants had relevant experience and could provide rich insights into the research questions [22,23]. This technique was selected for its ability to capture diverse perspectives, especially when exploring complex phenomena like health behaviors and engagement with services. By intentionally selecting participants based on their experiences with childhood weight management, this approach facilitated an in-depth exploration of themes emerging from the interviews.

### 2.5 Sample size

Qualitative research typically involves smaller sample sizes, focusing on the depth and richness of the data rather than the number of participants [24]. For this study, a sample size of 12–25 participants were recommended, allowing for a balance between diversity in perspectives and manageability for in-depth analysis. The final sample included 15 participants—eight parents and seven professionals. Data saturation was achieved when no new themes or insights emerged from the interviews, in line with the principle of data saturation in qualitative research [25,26]. The researchers initially gained access to participants through the HENRY (Health, Exercise and Nutrition for the Really Young) program in North East England. The Health, Exercise, and Nutrition for the Really Young (HENRY) program is a community-based intervention aimed at preventing obesity in preschool children [27,28]. It is implemented in various settings, including children's centers across the UK, and has also been adopted in other countries like Israel [29,30]. The program focuses on equipping parents with the skills and knowledge needed to foster healthier family lifestyles. Gatekeepers, such as program facilitators, were contacted to assist with identifying eligible participants. Due to difficulties in recruiting enough participants, the study was amended to extend recruitment across all of England. Additional participants were recruited from community physical fitness groups in Medway, South East England, through permission from group leaders.

### 2.6 Study procedures

Eligible participants were identified based on the inclusion criteria and approached through program facilitators or community group leaders. After obtaining informed consent, semi-structured interviews were conducted using open-ended questions designed to explore perceptions of childhood obesity and experiences with weight management services. Two interview guides were developed—one for parents and another for professionals. These guides were reviewed by experts to ensure they covered the relevant areas of interest, such as motivations for engaging with weight management services, barriers to participation, and perceptions of childhood obesity. The interviews were recorded and transcribed, with participants given the opportunity to review the transcripts for accuracy. Interviews lasted between 25 and 60 min and were conducted in a conversational style to create a relaxed environment where participants could speak freely.

### 2.7 Data collection and data management

Data was collected through semi-structured interviews, which allowed for flexibility in exploring emerging themes while ensuring that key topics were covered. The interviews were recorded, transcribed, and analyzed using thematic analysis, as outlined by Braun and Clarke [31]. Open-ended questions were used to elicit detailed responses from participants, focusing on their experiences, perceptions, and challenges related to childhood obesity and weight management programs. All data collected, including interview recordings, transcripts, and consent forms, were stored securely in accordance with the General Data Protection Regulations (GDPR) 2018. Only the researcher and supervisors had access to the data, which was stored on password-protected devices. Pseudonyms were used to ensure participant anonymity.

### 2.8 Data analysis

Thematic analysis was employed to analyze the data, following Braun and Clarke's [31] six-step process. This involved familiarizing with the data, generating codes, searching for themes, reviewing and refining themes, and producing a final report. Both inductive and deductive coding techniques were used to capture emergent themes related to the research questions, such as barriers to engagement, cultural influences on weight perception, and motivations for joining weight management programs. By employing thematic analysis, the study was able to systematically identify patterns in the data while remaining flexible enough to capture the complexities of participants' experiences and perceptions. Table 1 summarizes the methodological steps and processes used for the thematic analysis in the study.

**Table 1:** Key components of the thematic analysis method used in this study.

Component	Details
**Participants**	Two groups: Parents and Professionals. - Parents: 8 participants, varied in age, gender, ethnicity (Black African, White British), occupation status, and education level (no formal qualification, university degrees). - Professionals: 7 participants, included public health practitioners, GPs, and program facilitators.
**Data Collection**	Semi-structured interviews. - Interviews focused on motivations, challenges, benefits of weight management programs, socio-cultural influences, and perceptions of childhood obesity. - Audio-recorded and transcribed verbatim for accurate representation.
**Thematic Analysis Approach**	Braun & Clarke's [31] six-phase thematic analysis. - Familiarization with data - Generating initial codes - Searching for themes - Reviewing themes - Defining and naming themes - Producing the report
**Phase 1: Familiarization**	Immersing in data through repeated reading of transcripts and taking initial notes on key patterns.
**Phase 2: Generating Codes**	Initial coding of meaningful segments of data using descriptive labels. - Coded themes like “Barriers to Participation,” “Motivations for Joining,” “Cultural Beliefs on Weight."
**Phase 3: Searching for Themes**	Grouping related codes into broader themes. - Example themes: “Support from Family and Community,” “Barriers to Participation,” “Program Engagement."
**Phase 4: Reviewing Themes**	Themes were reviewed for accuracy, coherence, and clarity. - Some themes were merged (e.g., “Time Constraints” as a sub-theme under “Barriers").
**Phase 5: Defining & Naming Themes**	Each theme was defined, and sub-themes were included for clarity. - Final themes included: “Motivations for Joining the Program,” “Barriers to Participation,” “Cultural Influences on Weight Perception,” etc.
**Phase 6: Producing the Report**	Themes were integrated into a coherent narrative supported by participant quotes. Differences between parents and professionals were highlighted.
**Rigor & Trustworthiness**	Ensured by triangulation, peer review, and maintaining reflexivity.
**Final Themes Identified**	1. **Motivations for Joining** 2. **Barriers to Participation** 3. **Benefits and Positive Outcomes** 4. **Support from Family and Community** 5. **Socio-cultural Influences on Weight Perception** 6. **Program Engagement & Satisfaction** 7. **Consequences of Obesity**

## 3 Results

**Fig. 1:**
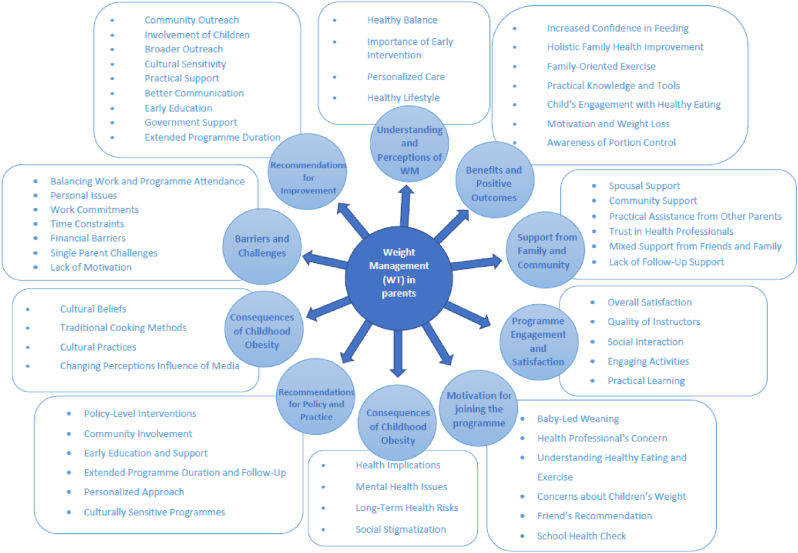
Detailed themes and sub-themes from parents' thematic analysis.

**Fig. 2:**
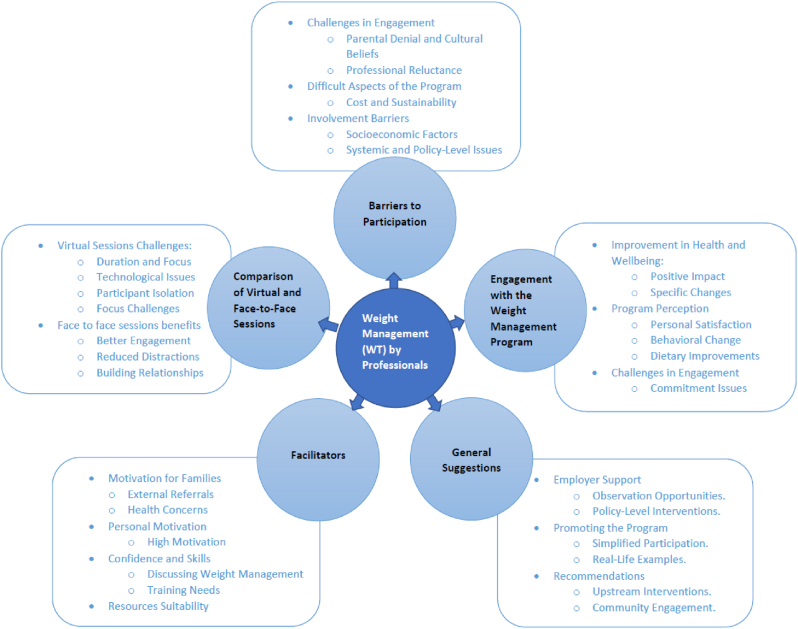
Detailed themes and sub themes from Professionals' thematic analysis.

The parent participants varied in age, gender, ethnic origin, occupation, and education level (See Table 2), while the professionals came from diverse roles such as community development workers, public health specialists, general practitioners, and program facilitators (See Table 3). This diversity provided a comprehensive understanding of the challenges and facilitators related to engaging families in weight management programs. The established themes for parents and professional participants are shown in Fig. 1, Fig. 2 accordingly.

**Table 2:** Sociodemographic details of respondents.

Respondents ID	Age	Gender	Ethnic Origin	Number of Children	Occupation Status	Education/Qualifications	Occupation
D-M	34	Female	Not specified	2	Unemployed	Not specified	Not specified
C–K	Not specified	Female	Black African (Malawian)	2	Full-time worker	University degree	Nurse
R–R	31	Female	White British	2	Full-time worker	University degree	Nurse
L-O	46	Female	Black, African	2	Full-time worker	University degree	Research Nurse
R-M	44	Female	Black, African	3	Full-time worker	University degree	Midwife
D-C	47	Female	African (Malawi)	1	Full-time worker	University degree	Social Worker
C-T	48	Female	African (Malawi)	1	Part-time worker	University degree (qualified in 2021)	Staff Nurse
S-A	46	Female	African	4	Not currently working	No formal qualification	Not specified

**Table 3:** Sociodemographic details of respondents.

Respondent ID	Age	Gender	Ethnic Origin	Occupation	Work Department	Occupation Status	Years in Current Role	Experience (Years)
NG	36	Female	White British	Community Development Worker	Ashington Children Center	Full-time	6 years	2 years (Henry program)
SS	43	Female	White British	Program Facilitator	Action for Children, Central Locality	Part-time	8 months	Previous early years programs
AP	–	Female	Not specified	Not specified	Not specified	Not specified	Not specified	Not specified
SL	43	Male	White British	Advanced Public Health Practitioner	Middlesbrough Council and Redcar Cleveland Council	Not specified	Not specified	20 years (Public Health)
AO	57	Female	Black African	Public Health Specialist	Kent County Council	Not specified	Not specified	Not specified
DM	52	Male	Black African	General Practitioner	Primary Care NHS	Full-time	10 years	Not specified
PK	40	Female	British Indian	Physician Associate	Primary Care	Part-time	3+ years	Not specified

### 3.1 Comparison of virtual vs. face-to-face sessions

• **Professionals' Perspective**: Professionals compared virtual sessions to face-to-face sessions and highlighted the limitations of virtual formats in terms of engagement, technological reliability, and interaction.

One professional noted, *“The virtual is just an hour session,”* (NG, 36) explaining that it shortened the depth of engagement and limited the material covered. Another pointed out the technical challenges: *“The computer or the Internet decided to go not today,”* (SS, 43) highlighting frequent interruptions. Professionals also mentioned the lack of interaction in virtual sessions: *“They were very isolated”* (SS, 43).

In contrast, face-to-face sessions were described as providing better engagement, with one professional stating*, “Parents can chat amongst themselves, and … you get a better flow of ideas”* (NG, 36). Face-to-face sessions also allowed families to build relationships: *“They build the relationships up with the other families”* (AP).

• **Parents' Perspective**: Although parents did not directly compare virtual and face-to-face sessions, they expressed a clear preference for engaging and interactive settings. One parent appreciated the opportunity to socialize: *“It was nice just to get out and speak to other adults as well … It's nice to associate with other adults who are going through the same situations”* (D-M, 34).

The structured activities, particularly for children, were also noted as beneficial: *“The programme was designed that the moms or the dads are also involved when the children are exercising”* (C–K, Female). This suggests that face-to-face sessions, which tend to facilitate such engagement, may be better suited for the kind of interactive learning that parents value.

### 3.2 Engagement with weight management programs

• **Professionals' Perspective**: Professionals emphasized that the effectiveness of the program depended heavily on parental engagement. One professional noted, *“The more the parents put in it, the more that the children get out of it,”* (NG, 36) reinforcing the importance of active participation. Improvements in children's eating habits were often directly linked to the parents' involvement: *"She's now started feeding in a different way”* (SS, 43).

However, life events often interrupted engagement. For example, illness and personal circumstances affected participation: *“Something comes along … illnesses or bereavement”* (NG, 36). A significant challenge mentioned was that some parents were in denial about their child's weight, which made it harder to engage them in the program: *“Parents do not recognize the issue, possibly because it's becoming a social norm”* (SL, 43).

• **Parents' Perspective**: Parents echoed similar experiences regarding the benefits of engagement. One parent explained how the program had increased her confidence in feeding her children: *“I'm a lot more confident now with feeding my son. I know what sizes to give them”* (D-M, 34). They also noted improvements in their children's lifestyle and physical activity: *“We're making a lot more healthier choices. And we're a lot more active now as well. We go for walks all the time”* (D-M, 34). However, personal circumstances, such as family illness, also posed challenges: *“This little one was really ill … Other than that, everything was always explained really well”* (D-M, 34). Parents faced practical barriers to consistent participation, including balancing work and family commitments: *“Balancing my work schedule with the program sessions was a challenge”* (D-M, 34).

### 3.3 Motivation and support

• **Professionals' Perspective**: For professionals, external referrals from health services and health concerns were key motivators driving family participation. As one professional explained, *“A lot of our referrals come from Children's services and health visitors”* (NG, 36). Health scares also acted as strong motivators for engagement: *“Health condition, it actually is a wake-up call for them”* (PK, 40). From their perspective, personal motivation to facilitate the program was high, particularly for those who believed strongly in its impact: *“My level of motivation is high for Henry … because I believe in the program”* (NG, 36).

However, some professionals expressed a lack of confidence in discussing weight with parents, pointing to gaps in their own training: *“If I had to specifically talk to a parent about their child's weight, I'm not sure that I would have the relevant tools”* (NG, 36).

• **Parents' Perspective**: Parents were motivated by both health professionals' concerns and personal factors. One parent noted that a health professional's concern about her child's weight initially caused defensiveness but later inspired her to engage: *“Initially I was like very defensive … but once I went for the first session I did realise that it was really important”* (C–K). Social influences, such as recommendations from friends, also played a role in participation: *“My friend commended me to join that programme”* (D-C, 47). Support from family members, particularly spouses, was crucial in maintaining engagement. One parent noted, *“Just my husband really, he is always supportive of anything I do … he read the books as well”* (D-M, 34). However, a lack of follow-up support from professionals was a barrier for some parents: *“There was no follow-up”* (D-C, 47).

### 3.4 Barriers to participation

• **Professionals' Perspective**: Professionals identified cultural beliefs, parental denial, and socioeconomic factors as key barriers to participation. Cultural perceptions of a healthy child being a “plump child” were seen as counterproductive: *“The belief that a healthy child is a plump child can be counterproductive for weight management service”* (AO, 57). Some professionals themselves were reluctant to address childhood obesity, with one stating, *“Our professionals are not recognizing that these children are at risk of obesity”* (SL, 43). Financial barriers also played a role, particularly for families from lower socioeconomic backgrounds: *“Families with a slightly lower social status … tend to be more defensive, less likely to seek help”* (DM, 52).
• **Parents' Perspective**: Parents faced practical challenges such as time constraints, financial difficulties, and balancing work and family life. One parent highlighted the high cost of certain healthy activities: *“Swimming … it's very expensive for the public to just take swimming lessons”* (R-M, 44). Single parents, in particular, struggled with balancing the demands of work and participation in the program: *“It was really difficult … especially for me as a single mum working at the same time”* (D-C, 47). Lack of motivation and visible results also discouraged some parents: *“Lack of motivation and sometimes when people are not seeing results”* (S-A, 46).

### 3.5 Recommendations for improvement

• **Professionals' Perspective**: Professionals recommended policy-level changes to make healthy choices more accessible and improve family support. One professional suggested, *“Making healthy choices easier choices for people … supporting families in terms of policy”* (AO, 57). Tailored support for families was also emphasized, with a recommendation for observation opportunities to enhance professional skills: *“Beneficial for me to observe”* (NG, 36).
• **Parents' Perspective**: Parents offered practical recommendations, including greater community outreach, more involvement of children in activities, and culturally sensitive programming. One parent suggested, *“I think if the children were more involved … it would motivate people to engage more”* (D-M, 34). Others called for culturally relevant adaptations to the program: *“They should be considerate of the culture as well, cause cultures are different”* (C–K). Parents also requested practical support, such as cooking classes: “Cooking classes would be great … like an after-school parent and child cooking class” (R–R, 31). Regular follow-up and extended program duration were also suggested: *“Weight loss is a long process … the program didn't last for long”* (C-T, 48).

## 4 Discussion

This study explored the perceptions and experiences of both professionals and parents engaged in weight management programs for children under five, uncovering several key themes that demonstrate the complexities involved in driving effective engagement. A central finding was the critical role of active parental involvement in the success of these programs. Both professionals and parents agreed that the effectiveness of weight management initiatives largely hinges on how deeply parents engage. Parents who actively participated in these programs observed significant improvements in their children's eating habits and levels of physical activity. This aligns with findings from Twiddy et al. [32], which emphasize that committed parental involvement is essential in promoting healthy behaviors in young children. Research by Kelleher et al. [33,34] further supports this, noting that parents are more likely to invest time and effort in these programs when they believe the outcomes—such as weight loss and improved emotional well-being—will significantly benefit their child.

However, the study highlighted numerous barriers to parental engagement. Cultural beliefs, in particular, were identified as obstacles, with the perception in some communities that a “plump” child is a healthy one. This misconception can result in parental denial of their child's weight issues and reluctance to participate in weight management programs, a challenge also noted by Parkinson et al. [35]. Similarly, practical challenges such as balancing work and family life, time constraints, and financial barriers—like the high costs of activities such as swimming—were cited by parents as reasons for inconsistent participation. These findings corroborate the work of Birch et al. [36] and Taveras et al. [37], who reported that logistical factors such as timing, location of sessions, and the availability of childcare can significantly impact program completion rates.

The study also explored the effectiveness of virtual versus face-to-face sessions, a topic of growing relevance since the COVID-19 pandemic accelerated the adoption of telehealth services. While virtual sessions provided convenience, both professionals and parents favored face-to-face interactions, noting that they foster better engagement, build stronger relationships, and create a sense of community among participants. This sense of community was seen as enhancing the program's overall effectiveness, as parents found motivation in connecting with others who shared similar experiences. Marsh et al. [38] similarly found that face-to-face interactions encourage more focused participation and allow parents to offer each other mutual support. The preference for face-to-face sessions is also supported by Baker et al. [39], who observed that participants were more likely to form trusting relationships with healthcare providers in person, facilitating open communication and greater adherence to program recommendations.

In contrast, virtual sessions, while convenient, were perceived as less effective due to technological challenges, reduced engagement, and difficulty maintaining focus—especially when parents had to simultaneously manage their children during the sessions. While virtual programs offer flexibility, these findings suggest they are less conducive to creating an interactive and supportive environment, limiting their long-term effectiveness. Kutz et al. [40] found that virtual sessions tend to be perceived as more informal, potentially lowering motivation levels. However, as suggested by Nuss et al. [41], a hybrid model that combines the accessibility of virtual care with the personal connection of face-to-face sessions may present the most effective solution for improving engagement in weight management programs.

Motivations for joining weight management programs varied between parents and professionals. Health concerns, particularly those raised by healthcare professionals, were a key motivator for parents. Many participants reported enrolling in programs after receiving advice from health visitors or following school health checks that highlighted concerns about their child's weight. This finding aligns with research by Moore et al. [11], which highlights the influence of direct referrals from trusted healthcare providers as a critical factor driving parental engagement. Persson et al. [42] also found that parents were more likely to participate when the recommendation came from a pediatrician or dietitian, emphasizing the importance of clear, proactive communication about the benefits of early intervention.

For professionals, motivation to deliver weight management programs effectively stemmed from a strong belief in their benefits. Many professionals expressed a deep sense of purpose in their work, believing that these programs could significantly improve children's long-term health outcomes if implemented well. This motivation is supported by Kelleher [43], who highlights that healthcare providers who believe in the program's potential are more likely to remain committed to its success, even when faced with challenges such as low engagement from families. However, some professionals in the study expressed the need for additional training and tools to effectively address sensitive topics such as childhood obesity, a sentiment echoed by Guell et al. [44] and Story et al. [45], who found that professional development and ongoing support are essential in ensuring healthcare providers feel equipped to tackle these complex issues.

Barriers to participation were another significant theme, with both parents and professionals identifying socioeconomic factors as critical obstacles. The cost of healthy food and access to recreational activities, such as swimming, were significant concerns, particularly for parents from lower-income backgrounds. These parents were often more defensive or hesitant to seek help, reflecting broader issues of stigma and access to resources. This finding aligns with Public Health England [46] and Taveras et al. [37], who noted that accessibility is a key factor in service uptake, particularly in deprived areas where transportation and time constraints are common barriers. Professionals also voiced concerns about the financial sustainability of running these programs, citing limited resources within health and social care services. This underscores the need for more robust support from policymakers to ensure that weight management programs remain accessible to families who need them, as also noted by Baker et al. [39].

The role of support systems emerged as crucial in facilitating parental engagement. Parents who had strong spousal support or were part of a supportive community were more likely to stay committed to the program and follow through with lifestyle changes. This finding supports research by Skouteris et al. [47] and Tomayko et al. [48], which emphasize the importance of social networks in sustaining participation. Furthermore, professionals recognized the importance of building trusting relationships with families, which was key to encouraging long-term engagement. Establishing a sense of community among participants was also seen as a way to reduce feelings of isolation and provide emotional and practical support, further reinforcing findings from Love et al. [49] and Baker et al. [39].

Finally, cultural and socio-cultural influences played a significant role in shaping how families approached weight management. Traditional cooking methods and cultural beliefs—such as the notion that a larger child is a healthier one—were seen as contributing factors to childhood obesity. This echoes findings by Jain et al. [50], who reported that parental misperceptions about weight are common, particularly among socioeconomically disadvantaged groups. Both professionals and parents emphasized the need for culturally sensitive approaches to weight management, with programs tailored to the beliefs and practices of different communities. Research by Guell et al. [44] and Barlow et al. [51] highlights the importance of cultural competence in healthcare, suggesting that culturally adapted interventions could significantly improve engagement and outcomes in weight management programs.

## 5 Limitations of the study

This study has several limitations that should be considered when interpreting the findings.

1. **Sample Size and Generalizability**: While the sample size of 15 participants allowed for in-depth qualitative insights, it limits the generalizability of the findings. The relatively small number of participants, particularly when divided into parents (8) and professionals (7), may not fully capture the broad range of experiences and perspectives across England.
2. **Regional Focus**: The study initially focused on North East England, with additional recruitment from Medway and South Tees areas. While these regions offer diverse socioeconomic contexts, they may not be representative of the entire country. Regional variations in cultural beliefs, socioeconomic status, and access to healthcare services may have influenced the findings, limiting the transferability to other regions of England or internationally.
3. **Recruitment Challenges**: The study encountered difficulties in recruiting a sufficient number of participants, leading to an expansion of the recruitment area. This may have introduced selection bias, as participants who were more engaged or aware of weight management programs might have been more willing to participate in the study. Consequently, the views of parents or professionals who are less involved or disengaged from such services might not be adequately represented.
4. **Self-Reported Data**: The data were based on self-reported experiences from both parents and professionals. As with all self-reported data, there is a risk of recall bias or social desirability bias, where participants may have provided answers, they perceived as socially acceptable or aligned with the researcher's expectations. This could particularly affect sensitive topics such as childhood obesity and weight management.
5. **Concerns of Physiological Factors and Health Outcomes:** The focus on children with a BMI above the 85th percentile acknowledges potential etiological variations in increased adiposity without including physiological reasons for each child's weight status. The study does not specifically examine individual physiological factors contributing to increased adiposity, as the scope is to understand perceptions and engagement with weight management services rather than medical diagnoses or direct health outcomes. The suggested outcomes from this study pertain to increased engagement and health awareness, rather than direct claims of weight reduction, with health improvements viewed as potential long-term benefits of increased service engagement and better understanding among caregivers and professionals.
6. **Virtual Interviews**: Due to logistical constraints, some interviews were conducted virtually via telephone or Microsoft Teams. While virtual interviews provide flexibility, they can limit non-verbal communication and may hinder rapport-building between the interviewer and participants, potentially impacting the depth and quality of the data collected. Face-to-face interactions might have elicited richer data, particularly on sensitive topics like weight management.

Addressing these limitations in future research could enhance the robustness of findings and improve the applicability of the results to a wider population.

## 6 Conclusion

The findings from this study underscore the intricate and multifaceted challenges faced by weight management programs for children under five. Both parents and professionals acknowledge the critical role these programs play in addressing early childhood obesity, yet several barriers persist that hinder effective engagement and long-term success. Key obstacles include cultural misconceptions about weight, parental denial, socioeconomic constraints, and systemic issues such as inadequate resources and professional training. These factors collectively contribute to the low uptake and inconsistent participation in weight management services, which limits their potential impact.

The study highlights the need for more tailored and culturally informed interventions. Many parents, especially in certain communities, hold beliefs that larger children are healthier, which can lead to delayed action or reluctance to engage in weight management programs. Addressing these deeply rooted cultural perceptions requires targeted outreach, education, and interventions that are respectful and responsive to cultural contexts. Additionally, creating an inclusive, supportive environment for families—one that includes the entire family unit in lifestyle changes—has been shown to improve both engagement and outcomes. From the perspective of professionals, the study reveals gaps in training and resources that affect their ability to effectively communicate the importance of childhood obesity interventions. Professionals need ongoing support and education to confidently discuss sensitive issues like childhood weight with families, ensuring they can foster trust and engagement.

To overcome these barriers, stronger systemic and policy-level support is essential. This includes increased funding for the sustainability of programs, particularly in disadvantaged areas, and the development of hybrid models that combine the convenience of virtual sessions with the engagement benefits of face-to-face interactions. The study demonstrates that a collaborative approach, where families, healthcare professionals, communities, and policymakers work together, is crucial to promote healthier behaviors and lifestyles for young children. This holistic effort will not only improve the immediate health outcomes of children but also help prevent long-term health complications associated with childhood obesity, contributing to a healthier future generation.

Based on the findings of this study, several recommendations emerge to improve engagement with and the effectiveness of weight management programs for children under five. These recommendations aim to address barriers, enhance participation, and ensure the sustainability of these programs.

• **Culturally Tailored Interventions**: Weight management programs should be culturally sensitive, taking into account the diverse beliefs, practices, and perceptions of health within different communities. This approach can help address cultural barriers, such as the belief that a larger child is healthier.
• **Hybrid Delivery Models**: Incorporating a hybrid model of both virtual and face-to-face sessions would improve accessibility while maintaining the benefits of personal interaction. Virtual sessions can provide convenience, while face-to-face meetings foster engagement and build stronger support networks.
• **Enhanced Professional Training**: Healthcare professionals delivering weight management programs should receive ongoing training on effective communication strategies, particularly around sensitive topics like childhood obesity. This will empower professionals to address these issues with confidence and empathy.
• **Increased Policy and Financial Support**: There is a need for stronger systemic support to ensure the financial sustainability of weight management programs. Policymakers should provide adequate funding and resources to maintain these services, especially in socioeconomically disadvantaged areas.
• **Family-Centered Approaches**: Engaging the entire family in the weight management process is critical for success. Programs should include family-oriented activities and promote peer support among parents to foster a sense of community and shared responsibility.
• **Follow-Up and Continued Support**: Long-term follow-up and continued support are necessary to ensure sustained behavior change. Programs should incorporate regular check-ins with participants, providing ongoing guidance and encouragement to maintain healthy habits.

## Ethical considerations

Ethical approval was obtained from Teesside University's Research Governance and Ethics Committee with the protocol number “065/19”. Written informed consent was obtained from all participants, who were fully informed of their rights, including the ability to withdraw from the study at any time. Participant confidentiality was strictly maintained, and all identifying information was removed from the data.

## Declaration of use of artificial intelligence

Artificial intelligence (AI) and AI-assisted technologies were not used in the writing or preparation of this manuscript.

## Data availability statement

The data that support the findings of this study are available upon request from the corresponding author.

## Authors’ contributions

MM conceived, designed and conducted the study; provided the research materials, reviewed, and wrote initial and final draft of the article. LAN supervised the study process; reviewed the first draft of the article; and provided logistic support. MJN provided logistic support; edited the final draft of the article; and interpreted data. SL provided logistic support; reviewed, and edited the final draft of the article. DBO provided logistic support, wrote initial and final draft of the article; analyzed, and interpreted data. All authors have critically reviewed and approved the final draft.

## Source of funding

This research received no specific grant from any funding agency, commercial or not-for-profit sectors.

## Declaration of competing interest

The authors declare that they have no known competing financial interests or personal relationships that could have appeared to influence the work reported in this paper.
